# Ecological Factors and Host Community Characteristics as Potential Drivers of Bat RNA Virus Spillover

**DOI:** 10.3390/biology15080609

**Published:** 2026-04-12

**Authors:** Jie Peng, Yuhang Liu, Chen Zhang, Hao Gu, Weihao Qi, Yutao Li, Fujie Han, Gang Liu, Mingxin Zhang, Xiaomin Yan, Kangkang Zhang, Ying Liu

**Affiliations:** 1 Jilin Provincial Key Laboratory of Animal Resource Conservation and Utilization, Northeast Normal University, 2555 Jingyue Street, Changchun 130117, China; 2 Jilin Provincial International Cooperation Key Laboratory for Biological Control of Agricultural Pests, Northeast Normal University, Changchun 130117, China; 3 Key Laboratory of Vegetation Ecology of Education Ministry, Institute of Grassland Science, Northeast Normal University, 5268 Renmin Avenue, Changchun 130024, China; 4 Key Laboratory of Cold Water Fish Germplasm Resources and Multiplication and Cultivation of Heilongjiang Province, Heilongjiang River Fishery Research Institute, Chinese Academy of Fishery Sciences, Harbin 150070, China; 5 State Key Laboratory for Diagnosis and Treatment of Severe Zoonotic Infectious Diseases, Key Laboratory for Zoonosis Research of the Ministry of Education, Institute of Zoonosis, and College of Veterinary Medicine, Jilin University, Changchun 130062, China

**Keywords:** virome, bat, spillover, ecological drivers, host community

## Abstract

Bats are reservoirs for many emerging viruses, yet broad-scale spatial patterns of bat viromes and their ecological determinants remain unclear, limiting spillover risk assessment. To address this, we conducted a standardized survey across central to southern China. During 2022–2023, fecal samples were collected from 527 bats representing 17 species at 21 caves in seven provinces. Using each cave as the analytical unit, samples from all species at a site were pooled to construct 21 fecal RNA virome libraries for metatranscriptomic sequencing; viral-like contigs were annotated, and viral alpha/beta diversity was quantified at the genus level. Sites were grouped by geographic distance and latitude to evaluate spatial differentiation and to relate virome patterns to host community attributes and environmental factors. We annotated 56 viral families, including 19 vertebrate-associated families, with multiple families and genera shared across geographic groupings. Vertebrate-associated viral diversity showed limited evidence of geographic isolation within the study region but suggested gradual turnover with latitude. Host species richness was the strongest correlate of virome diversity, exceeding the effects of evaluated environmental variables (e.g., climate and human activity). These results support prioritization of species-rich bat habitats for surveillance and risk assessment.

## Simple Summary

Bats are capable of harboring a wide array of viruses without exhibiting clinical disease, and certain of these viruses can occasionally spill over into other animal species or humans. Consequently, elucidating the drivers of such spillover events is critical for pandemic preparedness. In this study, we conducted a large-scale survey of bat-associated viruses across multiple regions of China to investigate spatial variation in viral assemblages and identify the key ecological and environmental factors underlying these patterns. Between 2022 and 2023, we collected fecal samples from 527 individual bats representing 17 species across 21 caves in seven provinces. Using metatranscriptomic sequencing, we characterized the viromes present at each site. Our analyses revealed a high diversity of viruses, including members of viral families known to infect mammals. While many viral taxa were shared among geographically distant sites—indicating limited spatial structuring by distance alone—we observed a gradual turnover in viral community composition along a latitudinal gradient, from lower to higher latitudes. Notably, the strongest predictor of increased viral diversity was local bat species richness, which exerted a greater influence than climatic variables or proxies for anthropogenic disturbance. These findings underscore the importance of prioritizing regions with high bat species diversity for enhanced viral surveillance and early-warning systems, thereby strengthening global capacities for outbreak prevention and response.

## 1. Introduction

Most emerging infectious diseases are zoonoses, posing substantial threats to global public health and the economy. Increasingly close interactions between humans and wildlife hosts have heightened the likelihood of animal-borne viruses spreading worldwide [1]. Therefore, characterizing viral communities in wildlife—together with their genetic diversity and ecogeographic distribution patterns—is of major importance, providing a scientific basis for preventing and controlling the transmission of Emerging Infectious Diseases (EIDs) originating from wildlife [2]. Current evidence indicates that bats are natural reservoirs for multiple high-impact EID viruses, such as coronaviruses and paramyxoviruses [3]. To strengthen preparedness for newly emerging zoonotic viruses, elucidating the range of factors that may influence bat virus spillover is a key foundation for risk assessment and the development of prevention and control strategies.

As highly social mammals, bats provide favorable conditions for pathogen transmission and persistence within populations. Bats are widely distributed and are the only mammals capable of true flight, which creates opportunities for close contact with humans and livestock across diverse ecological environments. Consequently, bats are regarded as natural reservoirs for many important zoonotic viruses [4]. Moreover, some bat species undertake long-distance flights during seasonal migration, which may accelerate viral spread and expand transmission ranges. To date, more than 4100 bat-associated animal viruses have been detected from 196 bat species across 69 countries worldwide [5,6]. Historically, bat pathogen surveillance has largely relied on targeted screening approaches for specific pathogens. Over the past two decades, multiple human viral disease outbreaks have been linked to bats, including severe acute respiratory syndrome coronavirus (SARS-CoV) [7], Middle East respiratory syndrome coronavirus (MERS-CoV) [8], severe acute respiratory syndrome coronavirus 2 (SARS-CoV-2) [9], Nipah virus (NiV) [10], and Ebola virus (EBOV) [10], all of which have had profound impacts on public health security and economic development. Accordingly, bats have become a major focus of virology research to facilitate the discovery of novel viruses and to evaluate the risks they pose for zoonotic spillover and subsequent transmission [11].

Although bats and their associated pathogens have been studied for decades, the field of bat viral ecology remains at a relatively early stage, and many key scientific questions still lack systematic investigation. Existing work has largely focused on leveraging virome data to characterize viral profiles across host taxa, discover novel viruses, and elucidate viral variation and evolution. For example, Wang et al. (2025) analyzed individual-level metatranscriptomes from 603 bats and identified 133 vertebrate-associated viral clusters, including nine clusters that are closely related to important human and livestock pathogens (e.g., SARS-related coronaviruses and SADS-related coronaviruses) [12]. However, ecological research on the factors that may influence spillover of bat-borne viruses and the mechanisms underlying these effects remains limited. Studies in other host groups suggest that climate variables are among the most frequently examined factors, yet their effects on viromes can differ across host systems. Higher temperature combined with lower relative humidity and precipitation has been associated with increased tick virome diversity [13], particularly by increasing the evenness of vertebrate-associated viruses, whereas rodent virome studies have reported negative associations between viral evenness and both precipitation and humidity [14]. In bat virome research, temperature and humidity have likewise been reported to be negatively correlated with virome diversity [15]. Beyond climatic drivers, other factors may shape host viral diversity differently across regions. For instance, a virome study of insectivorous mammals found that shrew viral diversity was higher at mid-elevations than at high elevations [16]. Within the limited body of work examining determinants of bat virome composition and diversity, variables such as host specificity, temperature, humidity, and human activities have been implicated. For example, Yan et al. (2025) analyzed the viromes of 40 bat taxa and found a negative association between human activity and virome diversity [15]. In addition, Wang et al. (2025) found that host specificity and geographic dispersion were important determinants of virome diversity in *Rhinolophus* bats [12]. Collectively, these findings suggest that the drivers of virome structure may vary across regions and species. Investigating factors influencing bat virus spillover across regions and spatial scales can therefore provide essential baseline data to advance mechanistic understanding of spillover and to improve early warning, prevention, and control of emerging infectious diseases.

China harbors high bat species diversity with a broad geographic distribution. However, current bat virome studies in China are spatially biased toward southern regions, particularly Yunnan Province, and the sampling effort has also preferentially focused on Rhinolophidae. For example, Li et al. (2024) investigated horseshoe bats (Rhinolophidae) from two sites in Hainan Province, China, and characterized the phylogenetic relationships and diversity of their mammalian viromes [17]. Peng et al. (2022) applied metaviromic approaches to *Rhinolophus sinicus* in Yunnan and detected sequences belonging to 22 viral families; the presence of Chikungunya virus (CHIKV), Getah virus, and Japanese encephalitis virus (JEV) was further confirmed by PCR [18]. Wang et al. (2023) used a metatranscriptomic framework to profile mammal-associated viruses in 149 bats from Yunnan, revealing high frequencies of viral co-infection (i.e., individual bats simultaneously infected with multiple viruses) and evidence of cross-species transmission among bats; based on phylogenetic relatedness to known pathogens and in vitro receptor-binding assays, they further identified five viral species with potential pathogenicity to humans or livestock [19].

Although a substantial number of bat virome studies have been conducted in China, differences in bat habitats and life-history traits among geographic regions may contribute to variation in virome composition and diversity [20,21]. However, constrained by host taxonomic coverage and sampling scope, our understanding remains limited regarding how host characteristics, ecological factors, and geographic location shape the composition and diversity of bat viromes. Therefore, sampling a broader range of bat species across more study sites, characterizing bat viral diversity and its spatial distribution patterns, and identifying potential drivers of viral spillover remain of critical importance. Building on previous work, we selected 21 bat roosting habitats to compare regional differences in the community diversity and composition of bat-borne viruses with spillover potential and to elucidate potential factors influencing bat viral spillover, with the aim of providing baseline data for a deeper understanding of spillover dynamics.

## 2. Materials and Methods

### 2.1. Sample Collection

**Figure 1:**
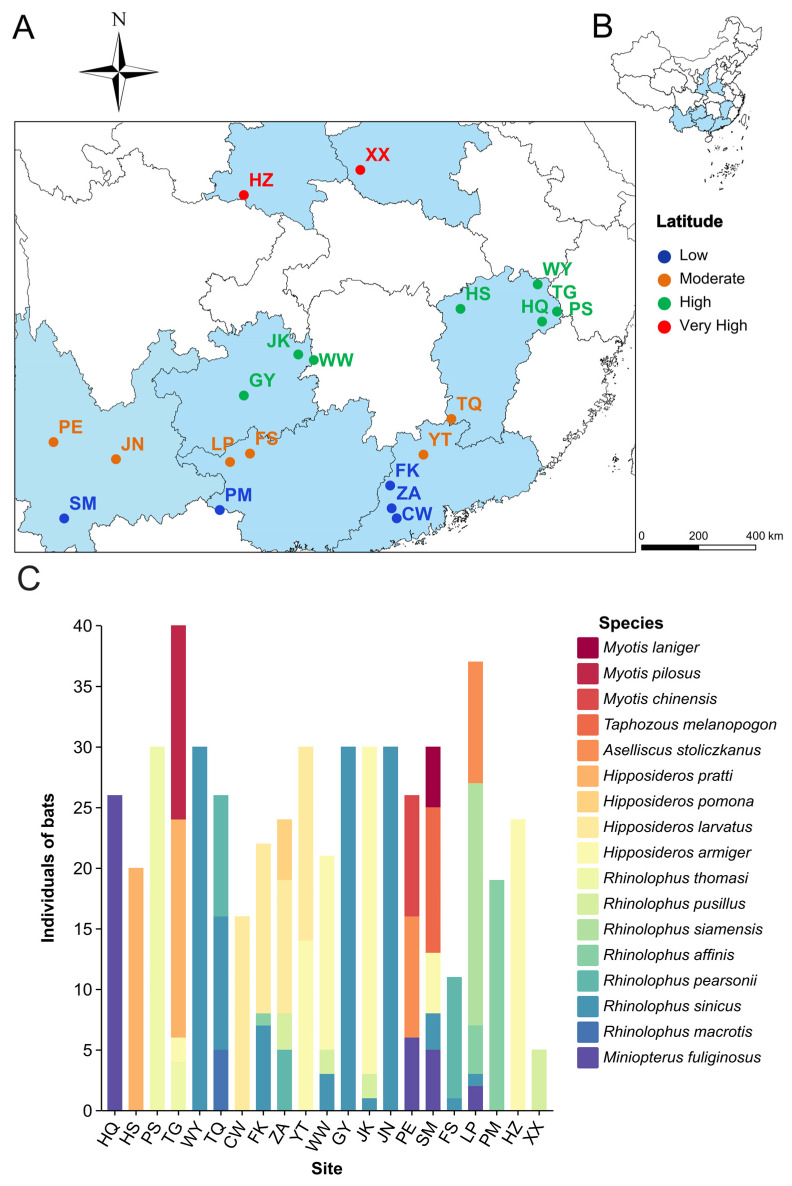
Distribution of sampling sites and sample collection numbers for bat virome. (**A**) Geographical locations of the 21 sampling sites (marked with solid points). (**B**) Provinces of the sampling sites, including PS, TQ, HS, WY, TG, HQ in Jiangxi Province; FK, ZA, CW, YT in Guangdong Province; GY, JK, WW in Guizhou Province; JN, PE, SM in Yunnan Province; FS, LP, PM in Guangxi Province; HZ in Shaanxi Province; XX in Henan Province. (**C**) Bat species composition and virome pooled-sample information at the sampling sites.

Bat samples were collected from 21 caves across China (Figure 1A), including PS, TQ, HS, WY, TG, and HQ in Jiangxi Province; FK, ZA, CW, and YT in Guangdong Province; GY, JK, and WW in Guizhou Province; JN, PE, and SM in Yunnan Province; FS, LP, and PM in Guangxi Province; HZ in Shaanxi Province; and XX in Henan Province (Figure 1B). The minimum distance between any two sampling caves was greater than 40 km, reducing the likelihood of overlap in bat movement among sampling sites. Each cave was sampled for 3–5 nights, and sampling was continued until no new bat species were captured so that the sampled bats broadly represented the species composition of each cave. Bats returning to the roost were captured using mist nets from 22:00 to 06:00 the next day. To avoid repeated sampling of the same individual, captured bats were marked by trimming hair on the back. Each captured bat was individually placed in a sterile paper bag and kept undisturbed for several hours to allow fecal collection. The fecal samples were then transferred into sterile 2 mL cryogenic tubes (Corning, Corning, NY, USA) and immediately frozen in liquid nitrogen in the field. To prevent cross-contamination between individuals, gloves were changed and sampling instruments were disinfected between collections. After all samples had been collected, they were transported to the laboratory on dry ice and stored at −80 °C. In total, fecal samples were obtained from 527 individuals representing 17 bat species, including *Rhinolophus sinicus* (*n* = 117), *R. macrotis* (*n* = 5), *R. pusillus* (*n* = 12), *R. pearsonii* (*n* = 25), *R. thomasi* (*n* = 34), *R. siamensis* (*n* = 20), *R. affinis* (*n* = 24), *Hipposideros larvatus* (*n* = 57), *H. armiger* (*n* = 88), *H. pratti* (*n* = 38), *H. pomona* (*n* = 5), *Aselliscus stoliczkanus* (*n* = 20), *Myotis chinensis* (*n* = 10), *M. pilosus* (*n* = 16), *M. laniger* (*n* = 5), *Taphozous melanopogon* (*n* = 12), and *Miniopterus fuliginosus* (*n* = 39). All sampled bat species were insectivorous. Among the bats collected, most individuals belonged to *Rhinolophidae* and *Hipposideridae*. Fecal samples were pooled by cave, yielding 21 virome libraries (see Table S1; Figure 1C). Sampling generally covered each cave, i.e., the bat species roosting at each sampling site were broadly represented. This study adhered to the ASAB/ABS Guidelines for the Use of Animals in Research. All experimental procedures in this study were approved by the Science and Technology Ethics Committee of Northeast Normal University, China (Approval No. 202301001, date of approval: 1 January 2023).

### 2.2. Geographic Clustering

To evaluate the effects of geographic distance on the composition and diversity of bat spillover viromes, we calculated great-circle (spherical) distances among sampling sites and clustered the 21 sites into six geographic regions using the R function hclust (complete linkage) [22]. The resulting groups were G1 (HQ, HS, PS, TG, WY), G2 (CW, FK, TQ, YT, ZA), G3 (GY, JK, WW), G4 (JN, PE, SM), G5 (FS, LP, PM), and G6 (HZ, XX).

To examine whether latitude and latitude-associated environmental factors influence the composition and diversity of bat spillover viromes, sites were further stratified into four latitudinal groups: Low, L (CW, FK, PM, SM, ZA), Moderate, M (FS, GY, JN, LP, PE, TQ, YT), High, H (HQ, HS, JK, PS, TG, WW, WY), and Very High, VH (XX, HZ). Cluster performance was assessed using the Silhouette algorithm based on the maximum great-circle distance among sites within each cluster. Silhouette scores range from −1 to +1, with higher values indicating better cohesion within the assigned cluster and clearer separation from neighboring clusters. The highest mean silhouette score among clusters was 0.7, suggesting good clustering performance and high reliability of the clustering results.

### 2.3. Viral RNA Extraction, Sequencing, and Virome Library Construction

Viral RNA extraction, sample processing, and virome library construction were performed with reference to previously described bat virome workflows [15,23]. To obtain an overview of the bat virome at each sampling site, fecal samples from all bat species collected at the same site were pooled and treated as a single sample for RNA metaviromic sequencing, generating 21 virome libraries (Figure 1C; detailed library information is provided in Table S1). Based on the number of bats captured per site, each pooled library comprised fecal samples from 5 to 40 individuals. Grouped fecal samples were transferred into 2 mL microcentrifuge tubes. For each pooled sample, 1 mL of pre-chilled (4 °C) 1× PBS was added, and the material was homogenized in a glass tissue grinder kept on ice throughout the procedure. The homogenate was collected into a 2 mL tube to obtain a fecal suspension, which was centrifuged at 4 °C and 1200 rpm for 10 min to separate the supernatant from the pellet. The supernatant was sequentially filtered through 0.45 µm and 0.22 µm membrane filters to obtain the filtrate. To minimize cross-contamination among sample pools during processing, areas of potential cross-contact were disinfected by spraying with ethanol. Pooling, homogenization, and filtration were all performed in a biosafety cabinet, thereby strictly preventing cross-contamination between fecal sample pools.

An aliquot (260 µL) of the filtrate was subjected to nuclease digestion, followed by lysis with three volumes of TRIzol for total RNA extraction. RNA concentration and purity were accurately quantified using a Qubit 2.0 Fluorometer (Invitrogen, Carlsbad, CA, USA) and a NanoPhotometer spectrophotometer (Implen, Munich, Germany). RNA integrity and potential DNA contamination were evaluated by agarose gel electrophoresis.

The workflow for constructing RNA virome libraries from bat fecal samples was as follows. First, ribosomal RNA (rRNA) was removed from total RNA that passed quality control using rRNA-targeting probes, yielding an rRNA-depleted (mRNA-enriched) RNA fraction. The resulting RNA was then randomly fragmented to ~250–300 bp in NEB Fragmentation Buffer using divalent cations. Using the fragmented RNA as template, first-strand cDNA was synthesized with random primers and dNTPs, followed by second-strand cDNA synthesis using the first strand as template. Sequencing adapters containing the P5/P7 sequences, index, and Rd1/Rd2 SP were ligated to the cDNA, and the target fragments were PCR-amplified and purified to generate the final sequencing libraries. Library quality was assessed using a Qubit 2.0 Fluorometer (Invitrogen, Carlsbad, CA, USA), an Agilent 2100 Bioanalyzer (Agilent Technologies, Santa Clara, CA, USA), and qRT-PCR to ensure library integrity and concentration. Libraries were further evaluated on a Qsep1 bio-fragment analyzer and sequenced on an Illumina NovaSeq 6000 platform (Illumina, Inc., San Diego, CA, USA) using paired-end 150 bp reads, with a minimum yield of 6 Gb of data per pool.

### 2.4. Annotation of RNA Virome Sequencing Data

For bioinformatic analysis of the sequencing data, to improve the accuracy of virome annotation and reduce noise, raw reads were first quality-filtered using fastp (v0.19.7) [24] to remove adapter sequences and low-quality reads. Host-derived reads were then removed by mapping against the bat reference genome using Bowtie2 (v2.4.1). Next, Kraken2 (v2.0.9-beta) was used to filter out reads assigned to bacteria, archaea, fungi, and other non-viral taxa. The remaining reads were assembled into contigs using metaSPAdes (v3.14.9). Assembled contigs were annotated against the EVRD database using BLASTn (v2.10.0) and DIAMOND BLASTx (v0.9.35). Results from nucleotide- and protein-level viral database searches were merged, and non-redundant sequences were extracted. These sequences were then queried against the GenBank non-redundant (nr) protein database to remove contigs annotated as non-viral, and the remaining bona fide viral sequences were retained. Indices were built for virus-associated contigs, and read counts mapped to these contigs were calculated using Bowtie2 (v2.4.1) and samtools (v1.10). Finally, together with online validation against GenBank, the final set of virus-like contigs (VLCs) was obtained.

### 2.5. Statistical Analyses

#### 2.5.1. Comparison of Viral Diversity and Geographic Distribution Patterns

In the analysis of bat RNA viromes, virus-like contigs (VLCs) were clustered using CD-HIT (v4.8.1) with the parameters similarity > 90% and short coverage > 80%. The resulting clusters were defined as viral operational taxonomic units (vOTUs). Indices were then built for these vOTUs, and read counts per vOTU were calculated by mapping reads with Bowtie2 (v2.4.1) and processing alignments with samtools (v1.10). Reads per million (RPM) were computed accordingly; to reduce false positives, only vOTUs with RPM > 1 were retained for downstream analyses. Based on these results, the number of reads and RPM values assigned to specific viral families, genera, and species in each sample were summarized, and figures were generated using ggplot2 in R (v4.1.4) [25].

Consistent with previous studies indicating that genus-level analyses yield virological inferences with biological and/or ecological relevance [26], we therefore characterized bat virome diversity and composition at the genus level. Alpha diversity (richness, Shannon index, and evenness) was calculated for each library as well as for the geographic-distance and latitude groupings using the vegan package in R (v4.1.4). Differences in alpha diversity among geographic and latitudinal groups were tested using the Kruskal–Wallis test implemented in the stats package. Beta diversity was quantified using Bray–Curtis dissimilarity and visualized by principal coordinates analysis (PCoA); significance was assessed with adonis2. Mantel tests (10,000 permutations) were used to evaluate the effects of geographic distance and host genetic distance on viral community composition (measured by Bray–Curtis dissimilarity). Pairwise geographic distances among sampling sites were computed using the rdist function in the fields package [27].

#### 2.5.2. Environmental Drivers of Vertebrate-Virus Diversity in Bat Viromes

To identify ecological factors associated with vertebrate-virus diversity in bat viromes (Shannon index, viral richness, and viral evenness), we fitted three independent sets of generalized linear models (GLMs) using the glm() function in the R package stats. Given the relatively small sample size, more complex models are more prone to overfitting and reduced generalizability; therefore, GLMs were used for the analysis. Each model set included all possible combinations of available and candidate predictors. Variables considered were the Human Footprint Index, forest area, cropland area, built-up (artificial surface) area, bat taxonomy, number of bat individuals pooled per library (hereafter referred to as sample size), bat sample diversity, mean monthly temperature, monthly precipitation, and monthly relative humidity. Human footprint (HFP) data were obtained from Mu et al. [28], which provides a global annual record of human pressures on terrestrial ecosystems at 1 km spatial and 1-year temporal resolution. The HFP index integrates eight anthropogenic pressure layers: built environments, population density, nighttime lights, cropland, pasture, roadways, railways, and navigable waterways. Each layer quantifies the intensity or presence of a specific human activity, and the combined index represents a gradient of human pressure across the landscape. Climate variables were obtained from the ERA5 hourly data on single levels from the 1940 to present dataset [29]. For the analyses, we extracted only the monthly climate data corresponding to each sampling event during 2022–2023. And land-cover data were derived from the global land-cover product based on a fine classification system [30]. Because datasets from different sources have differing spatial resolutions, all predictors were projected to the WGS84 coordinate system, and raster layers were resampled in ArcMap 10.8.1 using inverse distance weighting (IDW). A 10 km-radius buffer was generated around each sampling site, and the mean value of all pixels within the buffer was extracted for each variable. Multicollinearity among predictors was assessed using the vif() function in the car package, and only predictors with variance inflation factors (VIFs) < 5 were retained. The collinearity assessment was conducted on all candidate explanatory variables included in this analysis, namely Human Footprint Index, forest area, cropland area, built-up (artificial surface) area, bat taxonomy, number of bat individuals pooled per library, bat sample diversity, mean monthly temperature, monthly precipitation, and monthly relative humidity. After correlation assessment and VIF screening, the final set of predictors retained for model selection included bat taxonomy, number of bat individuals pooled per library, bat sample diversity, mean monthly temperature, monthly precipitation, and monthly relative humidity. Accordingly, the Human Footprint Index, forest area, cropland area, and built-up (artificial surface) area were excluded from the final model set. All candidate models were ranked by ΔAICc using the dredge() function in the MuMIn package [31], and the model with the lowest ΔAICc in each set was selected as the best-supported model.

Hierarchical partitioning was performed using the R package glmm.hp [32,33]. By calculating the independent contribution of each predictor within the model, we quantified the relative importance of ecological factors in explaining virome diversity. For GLM-based models, explanatory power was evaluated using McFadden’s R^2^ [34], whereas for GLMM-based models, it was assessed using the R^2^ metric implemented in glmm.hp. Marginal effects of predictors in the best-fitting model were estimated using the R package marginaleffects [35] and visualized with ggplot2 [25].

## 3. Results

### 3.1. Overview of Bat RNA Viromes

**Figure 2:**
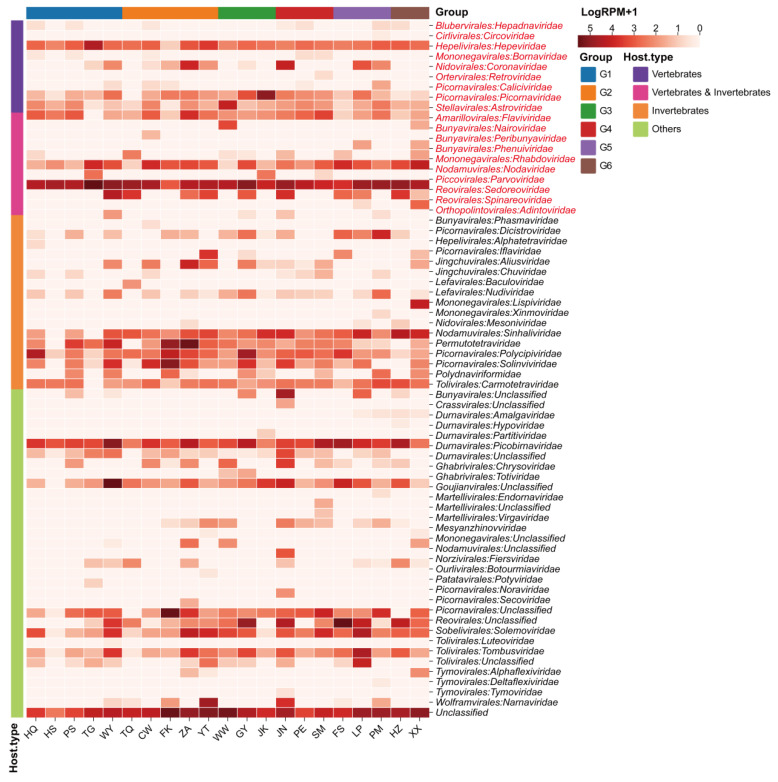
Virome Read Counts and Relative Abundance of Bat Guano. Heatmap generated based on normalized sequence reads of all viral families in each library, along with their sampling locations. The 21 libraries are arranged according to host type and geographical region. Names of viral orders and families are shown in the text lines on the right, with vertebrate-associated viruses highlighted in red.

From 2022 to 2023, samples were collected from 21 caves across seven provinces in China (Jiangxi, Guangdong, Guizhou, Guangxi, Shaanxi, and Henan), and 21 bat virome libraries were constructed by sampling site. Bat RNA viral metatranscriptomic profiling showed that the majority of viruses detected across the 21 libraries were RNA viruses, including invertebrate-, vertebrate-, plant-, and fungal-associated viruses, totaling 56 annotated viral families as well as several unclassified viruses (Figure 2). Several viral families were detected at all 21 sampling sites, including the invertebrate-associated *Iflaviridae*; the plant- and fungal-associated *Partitiviridae*; the plant-associated *Solemoviridae* and *Tombusviridae*; and the vertebrate-associated *Astroviridae*, *Hepeviridae*, *Nodaviridae*, and *Sedoreoviridae*.

Vertebrate-associated viruses comprised 19 families along with several unclassified viral sequences (See Table S2). With the exception of *Adintoviridae*, *Nodaviridae*, *Bornaviridae*, and *Circoviridae*, all other detected families include important pathogenic viruses (Figure 2). The most abundant vertebrate-virus families were *Sedoreoviridae*, *Picornaviridae*, *Nodaviridae*, *Spinareoviridae*, and *Hepeviridae*, with *Sedoreoviridae* showing the highest relative abundance. By contrast, *Circoviridae* exhibited the lowest relative abundance and was detected only in the PM cave. Across caves, the relative abundance of vertebrate viruses associated with bat spillover varied markedly, being highest in the TG cave and lowest in the FK cave. Notably, *Coronaviridae*, a family of high global concern, was detected at 57.14% (12/21) of the sampling sites (Figure 2).

### 3.2. Geographic Variation in Vertebrate-Associated Viral Diversity

**Figure 3:**
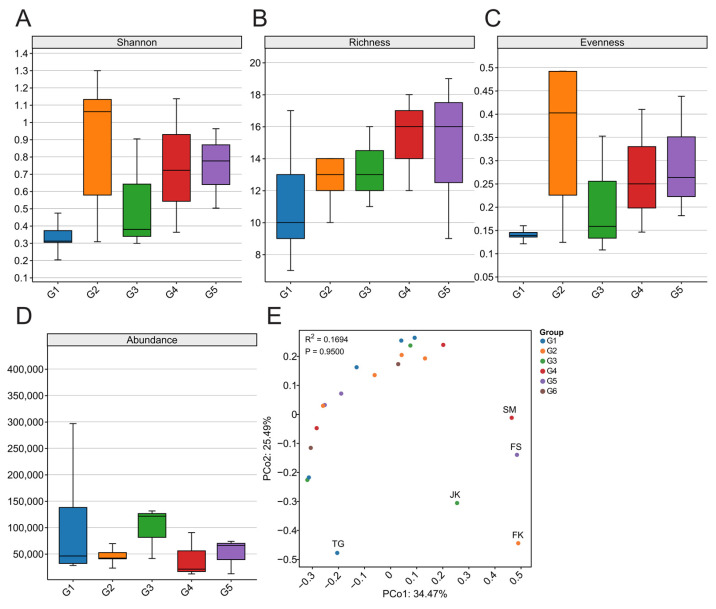
Differences in vertebrate-associated viral diversity in bats across geographical regions. (**A**) Shannon index; (**B**) Richness index; (**C**) Evenness index; (**D**) Abundance; (**E**) Principal coordinates analysis (PCoA) of the compositional structure of vertebrate-associated viruses in bats across different geographical regions.

Because viruses with zoonotic potential are predominantly vertebrate-associated viruses, we assessed viral alpha diversity (Shannon index, evenness, and richness) and beta diversity across sampling sites to evaluate the effects of geographic distance, latitude, and related environmental factors on viral community composition. In the distance-based grouping analysis, group G6 contained only two sampling sites and was therefore excluded from statistical testing. Kruskal–Wallis (KW) tests showed no significant differences in the alpha diversity or relative abundance of vertebrate-associated viruses among the five geographic clusters or among the three latitudinal groups (*p* > 0.05). To examine whether viral community composition (beta diversity) exhibited spatial structuring across geographic or latitudinal scales, principal coordinates analysis (PCoA) was performed on the Bray–Curtis dissimilarity matrix. The results indicated that beta diversity (i.e., viral composition) did not differ significantly among the five geographic groups or the three latitudinal groups (adonis2 test, *p* > 0.05; Figure 3E).

**Figure 4:**
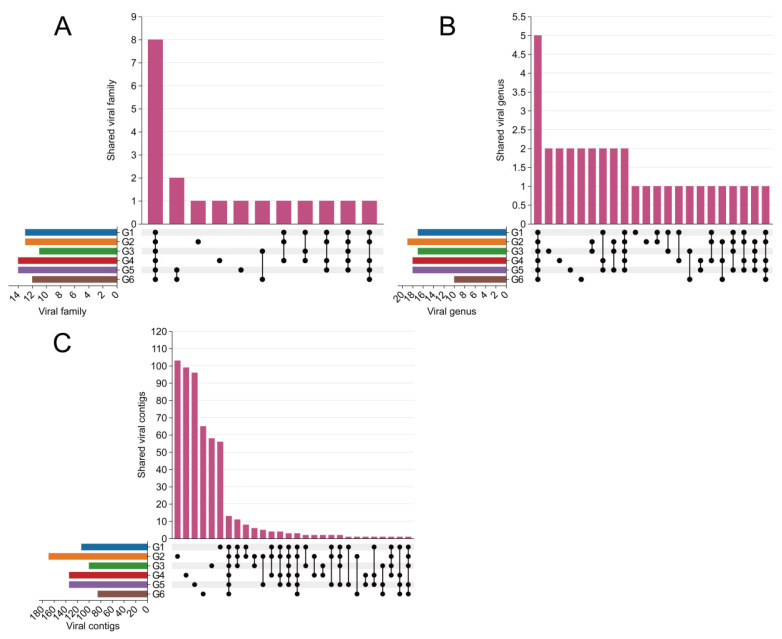
Shared Vertebrate-Associated Viruses Across Six Geographical Regions. Connected dots represent intersections, vertical bar plots indicate the number of viral taxa in each intersection, and horizontal bar plots show the total number of viral taxa in each geographical region. (**A**) Sharing of vertebrate-associated viruses at the family level across the six geographical regions; (**B**) Sharing of vertebrate-associated viruses at the genus level across the six geographical regions; (**C**) Sharing of vertebrate-associated virus sequences across the six geographical regions.

An UpSet plot was used to visualize virome overlap among the six geographic groups. Across the six groups, eight vertebrate-virus families and five genera were shared by all groups, accounting for 57.14–72.73% of vertebrate-virus families and 26.32–50.00% of vertebrate-virus genera detected within each group (Figure 4A,B). Thirteen zoonotic virus–related sequences were shared across all six groups, representing 7.69–15.29% of the total vertebrate-virus sequences in each group (Figure 4C). The maximum distance between the most distant sampling sites where these shared viral sequences were detected was 1878.69 km, indicating a broad geographic distribution and suggesting the potential for inter-site dissemination. Notably, the largest number of pairwise shared vertebrate-virus sequences was observed among the four sampling sites, CW, HQ, PE, and PS (27–28 shared sequences), most of which were assigned to *Flaviviridae* and *Hepeviridae*, implying potential cross-species transmission.

**Figure 5:**
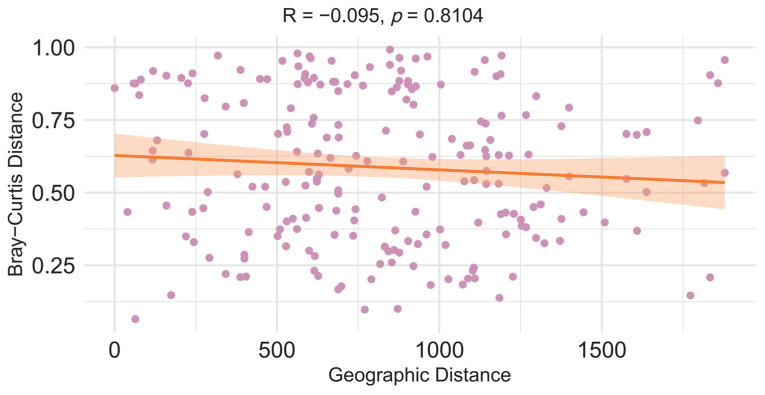
Correlation Between Vertebrate-Associated Viral Community Distance and Geographical Distance. This figure illustrates the comparison between Bray–Curtis distance of vertebrate-associated viruses in bat guano and the geographical distance of sampling communities.

To further characterize the geographic patterning of viral diversity, we examined the relationship between beta diversity and geographic distance. A Mantel test assessing the association between virome dissimilarity among sampling sites and pairwise geographic distance showed that geographic distance had no significant effect on vertebrate-virus community composition (Mantel R = −0.01, *p* > 0.05; Figure 5).

**Figure 6:**
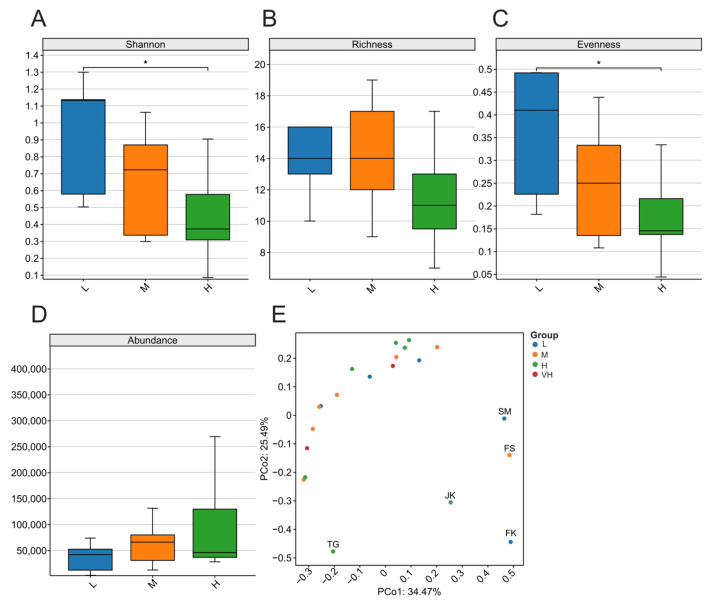
Differences in vertebrate-associated viral diversity in bats across geographical regions. (**A**) Shannon index; (**B**) Richness index; (**C**) Evenness index; (**D**) Abundance; (**E**) Principal coordinates analysis (PCoA) of the compositional structure of vertebrate-associated viruses in bats across different latitudinal zones. The latitudinal zones were defined as Low (L; CW, FK, PM, SM, ZA), Moderate (M; FS, GY, JN, LP, PE, TQ, YT), High (H; HQ, HS, JK, PS, TG, WW, WY), and Very High (VH; XX, HZ). * *p* > 0.05.

Due to the latitudinal span among sampling sites, we further investigated whether latitudinal differences influence the composition of spillover viruses. Sampling sites were divided into four groups according to latitude; because L4 contained only two sampling sites, no statistical analysis was performed for this group. The results indicated a trend toward higher viral diversity at lower latitudes (Figure 6). Specifically, the Shannon diversity index and evenness index differed significantly between the L1 and L3 groups (*p* < 0.05). However, Beta diversity did not differ significantly among the four latitudinal groups (adonis2 test, *p* > 0.05; Figure 6E).

### 3.3. Predictors of Bat Virome Diversity and Composition

The observed latitudinal trend in virome diversity is likely shaped by ecological factors that vary across latitudes. To further identify the predictors of vertebrate-associated viral diversity within bat viromes, we fitted generalized linear models (GLMs) and ranked candidate models using ΔAICc, with the model showing the smallest ΔAICc considered the best-supported. The results indicated that sample size and host diversity were key predictors of the Shannon index and Evenness index of vertebrate-associated viruses; temperature was included in the best-supported model for vertebrate-associated viral richness; and sample size was an important predictor of vertebrate-associated viral abundance. Overall, the model outcomes highlight the pivotal role of host diversity in structuring virome composition.

**Figure 7:**
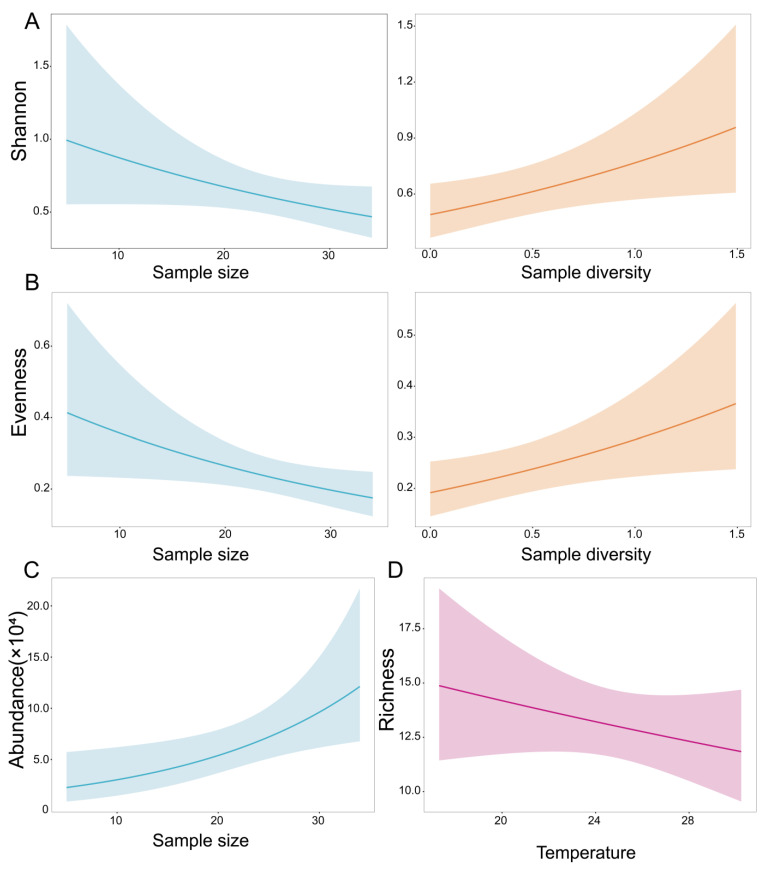
Partial Effect Plots for the Optimal Models of Vertebrate-Associated Viral Diversity. (**A**) Partial effect plot for the optimal model of viral evenness; (**B**) Partial effect plot for the optimal model of the Shannon index; (**C**) Partial effect plot for the optimal model of viral abundance; (**D**) Partial effect plot for the optimal model of viral richness.

**Figure 8:**
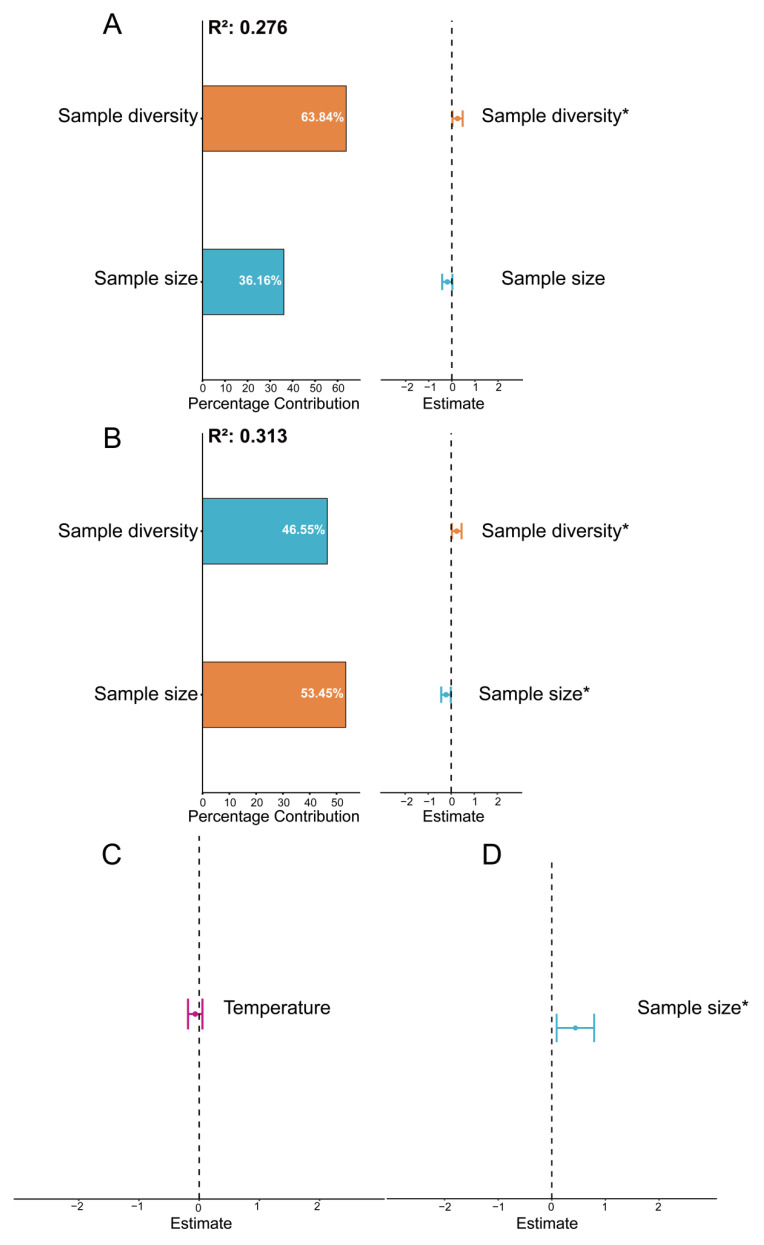
Optimal Models for Vertebrate-Associated Viral Diversity. Panels (**A**–**D**) show the optimal models for Shannon index, Evenness, Richness, and Abundance, respectively. The plots display the average parameter estimates (standardized regression coefficients) with their 95% confidence intervals, as well as the relative importance of each predictor (expressed as a percentage of explained variance) for all variables in the models. * *p* < 0.05.

Sample size (i.e., the number of bat individuals included in each pooled sample) and bat species diversity exerted distinct effects on vertebrate-associated viral community attributes. Sample size was significantly associated with multiple metrics of viral diversity. Specifically, sample size was negatively associated with the viral Shannon index and Evenness index (Shannon: *β* = −0.20, *p* > 0.05; evenness: *β* = −0.23, *p* < 0.05) (Figure 7A,B; Table 1). The best-supported model for the Shannon index explained 27.63% of the variance (Figure 8A), whereas the best-supported model for viral evenness explained 31.29% of the variance (Figure 8B), suggesting that increasing sample size may be accompanied by reduced viral diversity and a more uneven community structure, likely due to an increased dominance of common viral taxa within pooled samples. In contrast, viral relative abundance was positively associated with sample size (*β* = 0.44, *p* < 0.05), and the best-supported model explained 24.54% of the variance (Figure 8C), indicating that larger pools typically yielded higher viral abundance (Figure 8D). Meanwhile, sample diversity was positively associated with viral community attributes, including the Shannon index and Evenness index (Shannon: *β* = 0.25, *p* < 0.05; Evenness: *β* = 0.24, *p* < 0.05) (Figure 7A,B; Table 1), indicating that more compositionally diverse pools tend to harbor higher viral diversity and a more even distribution. However, most environmental covariates included in the models showed no significant effects on viral diversity or community attributes; only temperature showed a negative, but non-significant, association with viral richness (*β* = −0.07, *p* > 0.05) (Figure 7D; Table 1), and the best-supported model explained only 7.27% of the variance (Figure 8D).

To account for uncertainty in parameter estimates, reduce reliance on a single model, and avoid overlooking other potentially important predictors, we performed model averaging across the candidate models for the bat virome diversity prediction analyses (See Table S3). The predictors retained in the best-supported models varied among response variables, but mainly included sample diversity, sample size, temperature, and relative humidity. Their directions and magnitudes remained broadly consistent after model averaging (See Table S4), indicating that the results were relatively robust. The viral Shannon index was significantly and positively associated with sample diversity (*p* < 0.05, RI = 0.79) and negatively associated with sample size (*p* > 0.05, RI = 0.61). For Evenness index, the model-averaged results were almost identical to those of the best-supported model, showing a significant positive effect of sample diversity (*p* < 0.05, RI = 0.76) and a negative effect of sample size (*p* > 0.05, RI = 0.76). In the model-averaged results for viral richness, temperature had the highest relative importance (RI = 0.34), but its effect remained non-significant (*p* > 0.05); the relative importance of sample size and sample diversity was lower (RI = 0.16 and 0.27, respectively). Viral relative abundance was driven primarily by a significant positive effect of sample size (*p* < 0.05, RI = 1.00), whereas relative humidity showed a negative effect that did not reach statistical significance (*p* > 0.05, RI = 0.50).

## 4. Discussion

Based on metatranscriptomic sequencing, we characterized and compared the viromes of samples collected from 21 sites across China. In contrast to previous studies that largely focused on localized areas in southern China and on specific bat taxa [15,36], our sampling scheme expanded geographic coverage to underrepresented regions, including Jiangxi, Guizhou, Shaanxi, and Henan. This broader sampling provides a more accurate representation of the bat virome and allows for a comparative analysis of viral composition across different sites.

This study extends previous work by incorporating additional undersampled regions and bat species, thereby refining and strengthening the systematic characterization of bat viromes in China. Most viruses identified were widely distributed respiratory or enteric RNA viruses (e.g., astroviruses, coronaviruses, and reoviruses), consistent with previous reports [17], suggesting that the respiratory and gastrointestinal tracts are likely major routes of viral shedding in bats. Coronaviruses, in particular, pose substantial zoonotic risk. Experimental infection of fruit bats has shown that MERS-CoV replicates to higher levels in the respiratory tract, is shed from both the respiratory and intestinal tracts for up to 9 days [37], and that coronaviruses may exhibit tropism for bat respiratory and gastrointestinal tissues [38]. These findings further underscore the importance of prioritizing these two organs in future sampling efforts. Across the 21 sites, JN and WY yielded the highest numbers of vertebrate-virus sequence annotations. The dominant species at both sites was *Rhinolophus sinicus*. Previous studies have similarly reported that bats in the genus *Rhinolophus* harbor a greater diversity of RNA viruses, and our results further support the view that *Rhinolophus* bats may serve as key hosts for multiple mammalian viruses in China [21].

In our study, *Sedoreoviridae* was the most abundant viral family, consistent with the findings of Wang et al. (2023) from an individual-level bat virome study in Yunnan [19]. This pattern may reflect the enhanced environmental stability conferred by the protein architecture of *Sedoreoviridae* virions, which can help maintain infectivity under diverse environmental conditions and facilitate longer persistence and dissemination outside the host [39]. In addition, viruses in the family *Sedoreoviridae* are transmitted via the fecal–oral route [40], and thus are more readily enriched in fecal samples and appear at relatively high abundance, which may also increase exposure and transmission risk across hosts. Notably, *Sedoreoviridae* exhibits a broad host range: some members, such as rotaviruses (*Rotavirus*), are known to infect humans [41], whereas others (e.g., bluetongue virus, *Orbivirus*) are major pathogens of livestock [42]. Collectively, the detection of *Sedoreoviridae* in bat viromes suggests that bats may contribute to the maintenance and dissemination of viruses with potential for cross-host transmission across regions.

Many vertebrate-associated viruses detected in this study showed broad distributions and represent commonly reported viral taxa. For example, members of *Astroviridae*, *Hepeviridae*, *Nodaviridae*, and *Sedoreoviridae* were present at all sampling sites. This widespread occurrence may also reflect interregional dissemination driven by bats’ flight capability.

The PCoA showed partial clustering among several sampling sites, suggesting some similarity in virome composition among those sites. Examination of the original abundance matrix indicated that these samples shared several recurrent dominant viral taxa, particularly *Orthoreovirus* and other common virome components. In contrast, several sites were more dispersed in ordination space, suggesting greater virome heterogeneity. This pattern may be related to site-specific enrichment of particular viral taxa, such as *Shanbavirus* at JK and high *Orthoreovirus* abundance at TG (see Table S2). Differences in environmental variables and host family may also have contributed to the separation of these sites. For example, JK and FS were associated with relatively high forest cover and low water availability, whereas TG was characterized by higher temperature, lower precipitation, and higher agricultural and water-related indices. In addition, SM differed from most other sites in being dominated by Emballonuridae, while FS was dominated by Rhinolophidae and JK/TG/FK by Hipposideridae, suggesting that host composition may also have influenced virome structure. Together, these results suggest that both local viral enrichment and ecological differences among sites may explain why some locations did not cluster with the others.

Across the six geographic distance groups, we observed no significant differences in vertebrate-associated viral diversity, and geographic distance was not significantly associated with dissimilarity among groups. These results indicate that, within the spatial extent of this study, the bat virome did not exhibit a clear pattern of geographic structuring. Although geographic distance did not explain significant variation, latitude-based grouping suggested higher viral diversity at lower latitudes. For example, L (low latitude) and M (mid latitude) differed significantly in Shannon diversity and evenness (*p* < 0.05). However, when comparing latitude groups overall, most metrics did not reach statistical significance (*p* > 0.05), implying that latitude may influence viral diversity in a continuous, gradual manner rather than generating distinct biogeographic partitions. Because latitudinal differences also reflect variation in ecological factors such as temperature, we further examined other potential drivers. Accordingly, we applied a multifactor model to evaluate the effects of host type, pooling strategy, and ecological variables on virome diversity.

The results suggest that latitude-associated environmental gradients (e.g., temperature) may have a potential influence on viral richness, but the statistical support was weak. In the best-fitting model, monthly temperature was negatively associated with viral richness (*β* = −0.07), yet this relationship was not statistically significant (*p* = 0.281). Previous studies have highlighted the ecological importance of temperature and related climatic factors in shaping animal virome diversity. However, the pattern observed in our study should be interpreted with caution, as differences in bat species distribution across sampling sites may also have contributed to variation in viral richness. Because some bat species were not uniformly distributed across the sampling area and we did not explicitly assess the effect of host species composition, this remains a potential confounding factor. For instance, Yan et al. (2025), based on 8176 bat RNA virus metagenomic datasets, reported significant negative correlations between temperature, humidity, and bat virome diversity, suggesting that viral communities in warmer or more humid environments may experience stronger environmental filtering or host niche constraints [15]. This pattern is directionally consistent with the negative association between temperature and viral richness observed in our study. In addition, the weak temperature effect may also be related to the uneven geographic coverage of our sampling. Most samples in this study were collected from southern China, whereas northern localities were underrepresented, and some northern provinces were excluded from parts of the analysis because of limited sample size. As a result, the climatic gradient represented in our models may have been narrower than the full environmental gradient across China, reducing our power to detect broad-scale temperature–virome relationships. Therefore, the observed temperature effect should be interpreted with caution. Future studies with broader latitudinal coverage and more balanced sampling across climatic zones will be necessary to better evaluate the role of temperature in shaping bat virome diversity. Mechanistically, temperature may influence animal virome diversity through multiple pathways. On the one hand, higher temperatures may reduce the stability of some viruses outside the host or in the environment [43], thereby constraining their long-term persistence; on the other hand, temperature can indirectly reshape viral transmission networks by modulating host immune function [44], population density, and behavioral patterns [14].

Given that our sampling was conducted exclusively during the summer season, we employed monthly average temperature, precipitation, and relative humidity corresponding to the sampling period rather than long-term bioclimatic variables (e.g., temperature seasonality or annual temperature range). These short-term meteorological variables provide a more direct snapshot of the immediate environmental conditions associated with viral replication, shedding, and detection at the time of sampling [45,46]. In contrast, long-term climatic variables such as temperature seasonality or annual temperature range may be more appropriate for studies based on long-term longitudinal monitoring and repeated sampling, as they are better suited to capturing broader ecological processes underlying viral circulation, persistence, and host adaptation. In future work, it is necessary to conduct long-term monitoring and sampling and integrate long-term climatic variables to further investigate the ecological factors shaping bat virus transmission and maintenance [47,48].

In contrast, under the pooling strategy, sample size and within-sample species diversity were more influential in explaining virome Shannon diversity and evenness: virome Shannon diversity and evenness decreased with increasing sample size but increased with higher within-sample species diversity. In our study, the number of bat individuals pooled per library (sample size) may, to some extent, reflect the size and composition of the bat assemblage within a cave. Therefore, the negative association between sample size and both Shannon diversity and viral evenness may suggest that larger bat assemblages or cave systems tend to harbor a more homogeneous viral community composition, in which a few dominant viral taxa are repeatedly detected across individuals and become disproportionately represented. Ecologically, this may indicate that in larger bat colonies, commonly shared viruses are more likely to persist and circulate among hosts, thereby dominating pooled viromes, reducing community evenness, and lowering diversity indices that are sensitive to relative abundance distributions. Chen et al. (2023) similarly found that sample size is an important determinant of viral community composition [20], underscoring the need for standardized sampling strategies in viral ecological surveys. Future work should prioritize optimizing sample size to improve comparability across virome datasets. Moreover, because we sampled essentially all bat species roosting in each cave and constructed each pooled sample in proportion to the number of individuals captured for each bat species, the pooling scheme reflected the bat species composition and species richness of the sampled caves. Therefore, our model results, to some extent, capture the effects of host diversity and richness on viral spillover. Studies in other animal taxa have also shown that viral diversity can, to a certain extent, track host richness. For example, when pooling samples by host group (e.g., rodents, bats, shrews), a larger number of hosts typically covers more host lineages and ecological niches, increasing the likelihood of detecting a broader set of viruses; consequently, host number is often positively associated with viral diversity [3,20]. Mollentze et al. (2020) further reported a significant positive relationship between zoonotic virus diversity and host diversity in mammals [49]. Our findings therefore suggest that, within bats, higher local bat species diversity may be associated with greater diversity of spillover-prone viruses. This highlights the value of prioritizing bat habitats with high species diversity in viral surveillance and prevention efforts.

Beyond local host diversity, variation in bat species composition among sampling sites may also contribute to spatial patterns of viral overlap. For example, WW and JK, separated by 58.1 km, shared three bat species (*Rhinolophus sinicus*, *R. pusillus*, and *Hipposideros armiger*) and also exhibited multiple shared viral taxa. This result suggests that viral overlap among geographically nearby sites may be partly associated with shared host species and local host movement. Shared viral taxa were also detected among more distant site pairs with overlapping bat taxa, such as GY and JN (502.0 km; both containing *R. sinicus*) and FS and TQ (717.0 km; both containing *R. sinicus* and *R. pearsonii*). Although these bat species are not known to undertake long-distance migration, their populations may still expand through short-distance dispersal and stepwise movements among neighboring habitats, which could promote virus transmission between sites and potentially contribute to the occurrence of shared viruses across more distant habitats. However, this inference remains speculative and requires further investigation to be confirmed. This broader-scale overlap may be consistent with a role for host dispersal or roost-switching behavior, although it may also reflect the wide distribution of some viruses or the presence of unsampled intermediate habitats linking apparently distant sites. Published studies suggest that bats in the families Rhinolophidae and Hipposideridae are not typical long-distance migrants, but they may undergo seasonal movements and roost switching [50,51], including separation of summer and winter roosts, local or regional shifts among roosts, and in some cases altitudinal movements [52]. In addition, studies have shown that some *Rhinolophus* species may move up to 30 km for hibernation [53]. These behaviors may facilitate viral exchange among colonies. Therefore, viral sharing among sites may be influenced not only by local host richness and taxonomic overlap but also by host movement and seasonal changes in roost use.

This study also has limitations. Our analysis focused exclusively on RNA viruses and did not include DNA viruses, which are also important components of bat viromes; future studies incorporating both RNA and DNA viruses would provide a more comprehensive picture of bat-associated viral diversity. We pooled samples at the sampling-site level prior to sequencing and characterized the bat virome for each site; consequently, our data cannot resolve differences in virome diversity and abundance among individual bats or among bat species. In addition, given that bat community composition varies across sampling regions and is itself shaped by geographic, topographical, and bioclimatic conditions, the regional patterns observed in this study may partly reflect differences in host composition rather than independent regional effects. Ideally, the influence of geography on virome composition would be better evaluated by comparing different geographic populations of the same bat species, but our study was designed to compare virome composition among bat communities at different sites using pooled samples; the current dataset does not support species-level virome comparisons. Nevertheless, the regional grouping and pooled-site comparisons still suggest that virome composition among bat communities may be shaped jointly by geographic setting and host community composition [54,55]. Increasing sampling resolution by conducting virome surveillance and comparisons at the individual or population scale would better enable evaluation of the frequency and determinants of viral spillover and transmission among bat individuals and populations and would also help reduce the influence of potential confounding effects [19]. Nevertheless, we detected a broad set of common bat-associated viral families, providing a relatively comprehensive overview of viral diversity within bat communities. Compared with individual- or population-level virome profiling, this site-level pooling approach is less costly and is therefore more feasible for future large-scale, long-term viral surveillance, offering early warning signals and an evidence base for the prevention and control of emerging infectious diseases.

## 5. Conclusions

This study demonstrated that, across the central–southern China study transect, vertebrate-associated viruses in bats did not exhibit pronounced geographic partitioning among sites but may instead vary gradually along a latitudinal gradient. Host community diversity was identified as the dominant factor shaping bat virome diversity, with effects that exceeded those of the assessed environmental covariates, including climate and indices of human influence. These findings imply that habitats harboring high bat species richness should be prioritized in surveillance programs and spillover-oriented risk evaluation for emerging infectious diseases. In addition, by contributing baseline data on bat viromes in China, this work outlines a feasible, relatively low-cost approach for large-area monitoring, offering a foundation for sustained early warning and future prevention and control efforts.

## Figures and Tables

**Table 1:** Summary of Optimal Models.

Model 1: Virome Shannon ~ Sample Size + Sample Diversity					
Predictors	Estimate	Std. Error	t value	Pr (>|t|)	*p*-value adj
(Intercept)	−0.49	0.11	−4.49	<0.001	
Sample Size	−0.20	0.11	−1.75	0.097	0.146
Sample Diversity	0.25	0.11	2.20	0.041	0.049
Model 2: Virome Evenness ~ Sample Size + Sample Diversity					
Predictors	Estimate	Std. Error	t value	Pr (>|t|)	*p*-value adj
(Intercept)	−1.44	0.10	−13.81	<0.001	
Sample Size	−0.23	0.11	−2.12	0.049	0.084
Sample Diversity	0.24	0.11	2.24	0.038	0.062
Model 3: Virome Richness ~ Temperature					
Predictors	Estimate	Std. Error	z value	Pr (>|z|)	*p*-value adj
(Intercept)	2.57	0.06	42.4	<0.001	
Temperature	−0.07	0.06	−1.08	0.281	0.281
Model 4: Virome Abundance ~ Sample Size					
Predictors	Estimate	Std. Error	t value	Pr (>|t|)	*p*-value adj
(Intercept)	11.11	0.17	63.81	<0.001	
Sample Size	0.44	0.18	2.467	0.023	0.023

## Data Availability

The data on which this study is based are available in the Supplementary Material. RNA sequencing raw data were deposited into CNGB Sequence Archive (CNSA) of China National GeneBank database (CNGBdb) under accession number CNP0008443.

## Supplementary Materials

The following supporting information can be downloaded at https://www.mdpi.com/article/10.3390/biology15080609/s1, Table S1: Bat virome samples pooled by cave; Table S2: Taxonomic information and the relative abundance of viruses identified in sampling sites; Table S3: Candidate Models; Table S4: Summary of model averaged.

## Abbreviations

The following abbreviations are used in this manuscript:

HQ	Hongqiao
HS	Huangsha
PS	Paishan
TG	Tiangui
WY	Wuyuan
TQ	Tuqi
CW	Chunwan
FK	Fengkai
ZA	Zhenan
YT	Yantou
WW	Wawu
GY	Guiyang
JK	Jiangkou
JN	Jinning
PE	Puer
SM	Simao
FS	Fengshan
LP	Langping
PM	Pingmeng
HZ	Hanzhong
XX	Xixia
